# In vivo kinematics and cruciate ligament forces in bicruciate-retaining total knee arthroplasty

**DOI:** 10.1038/s41598-021-84942-y

**Published:** 2021-03-11

**Authors:** Kenichi Kono, Hiroshi Inui, Tetsuya Tomita, Takaharu Yamazaki, Shoji Konda, Shuji Taketomi, Sakae Tanaka, Darryl D. D’Lima

**Affiliations:** 1 Department of Molecular Medicine, The Scripps Research Institute, La Jolla, CA 92037 USA; 2 Department of Orthopaedic Surgery, Faculty of Medicine, The University of Tokyo, 7-3-1 Hongo, Bunkyo-ku, Tokyo 113-0033 Japan; 3 Department of Orthopaedic Biomaterial Science, Osaka University Graduate School of Medicine, 2-2 Yamada-oka, Suita, Osaka 565-0871 Japan; 4 Department of Information Systems, Faculty of Engineering, Saitama Institute of Technology, 1690 Fusaiji, Fukaya, Saitama 369-0293 Japan; 5 Department of Health and Sport Sciences, Osaka University Graduate School of Medicine, 2-2 Yamada-oka, Suita, Osaka 565-0871 Japan; 6 Shiley Center for Orthopaedic Research and Education at Scripps Clinic, La Jolla, CA 92121 USA

**Keywords:** Health care, Medical research, Risk factors

## Abstract

We analyzed the effects of bicruciate-retaining total knee arthroplasty (BCR-TKA) on knee kinematics and cruciate ligament forces. Patients (N = 15) with osteoarthritis (OA) and an intact anterior cruciate ligament (ACL) underwent magnetic resonance imaging and single-plane fluoroscopy to measure tibiofemoral kinematics during two deep knee bend activities before and after BCR-TKA: (1) weight-bearing squat; (2) non-weight-bearing cross-legged sitting. Forces in ligament bundles were calculated using VivoSim. The dynamic range of varus-valgus angulation decreased from 3.9 ± 4.4° preoperatively to 2.2 ± 2.7° postoperatively. Preoperatively, the medial femoral condyle translated anteriorly from 10° to 50° of flexion, and posteriorly beyond 50° of flexion. Postoperatively, the medial and lateral femoral condyles translated posteriorly throughout flexion in a medial pivot pattern. ACL forces were high in extension and decreased with flexion pre- and postoperatively. PCL forces increased with flexion preoperatively and did not change significantly postoperatively. Preoperatively, ACL forces correlated with anteroposterior translation of the femoral condyles. Postoperatively, PCL forces correlated with anteroposterior translation of the lateral femoral condyle. BCR-TKA altered knee kinematics during high flexion activity which correlated significantly with changes in cruciate ligament forces.

## Introduction

Osteoarthritis (OA) induces significant changes in knee kinematics^1,2^. While total knee arthroplasty (TKA) is successful in relieving pain and partially restoring function, cruciate-retaining, cruciate-sacrificing, and cruciate-substituting TKA designs do not restore normal knee kinematics^3^. These abnormal kinematics have been attributed to the sacrifice of the anterior cruciate ligament. Bicruciate-retaining total knee arthroplasty (BCR-TKA) designs attempt to recreate normal knee movement by preserving both the anterior cruciate ligament (ACL) and the posterior cruciate ligament (PCL). Studies have found that knees that underwent BCR-TKA exhibited kinematics and stability similar to normal knees^4–7^. A few studies have also reported favourable patient satisfaction and long-term survivorship and function of knees after BCR-TKA^8–10^. However, not all reports have been universally positive. Studies have reported that clinical outcomes after BCR-TKA did not differ from the outcomes reported for knees that underwent ACL-sacrificing TKA^11,12^. Moreover, several studies demonstrated that knee kinematics during treadmill walking after BCR-TKA were not the same as normal knees and that anterior cruciate ligament forces were higher in cadavers implanted with BCR-TKA^13,14^. However, the change in knee kinematics and cruciate ligament force after BCR-TKA is largely unknown. Moreover, the relationship between knee kinematics and cruciate ligament force remains unknown.

Many people desire the ability to perform high knee flexion activities such as squatting, kneeling, gardening, sitting on the floor, or practicing yoga. Additionally, studies have linked high knee flexion function to clinical outcomes, patient satisfaction, or expectation after TKA^15,16^. Therefore, evaluating high knee flexion activities is clinically relevant. A previous study demonstrated that knee kinematics after BCR-TKA differed depending on the type of high-flexion activities^17^. However, whether the cruciate ligament force in BCR-TKA knees differs depending on the high-flexion activities remains unknown.

This study was designed to analyze the effects of BCR-TKA implantation on knee kinematics and cruciate ligament force during high knee flexion activities and to determine if knee kinematics were linked to cruciate ligament forces. We therefore studied two types of high flexion activities: a closed-kinetic chain deep knee bend (squatting) and an open-kinetic chain high flexion (sitting cross-legged). Our null hypothesis was that implantation with a BCR-TKA and the type of high knee flexion activity would not change kinematics or ligament forces in the cruciate ligaments.

## Results

All components of KOOS increased significantly postoperatively; HKA angle also increased from 172° to 178° (Table 1).

**Table 1:** Knee injury and osteoarthritis outcome scores (KOOS) and hip-knee-ankle angle (HKA).

	Preoperatively	Postoperatively	*p*-value
**KOOS (points)**			
Pain	44.8 ± 15.9	81.9 ± 6.6	≤ 0.01*
Symptoms	54.6 ± 11.7	78.3 ± 9.9	≤ 0.01*
Function in daily living activities	54.0 ± 16.4	82.0 ± 6.7	≤ 0.01*
Function in sports and recreation	15.4 ± 17.3	44.7 ± 21.4	≤ 0.01^a^
Quality of life	23.2 ± 15.0	62.9 ± 17.1	≤ 0.01*
**HKA (degrees)**	171.8 ± 4.9	178.2 ± 1.7	≤ 0.01*

*A paired t-test.

^a^Wilcoxon signed-rank test.

### Kinematic changes

Preoperatively knee flexion ranged from 0.3 ± 8.3° to 118.7 ± 12.4° during squatting. Postoperative knee flexion ranged from –4.7 ± 4.3° to 115.7 ± 12.4° during squatting and from –1.7 ± 2.3° to 115.7 ± 12.4° during cross-legged sitting. Maximum knee extension, but not knee flexion, increased significantly postoperatively (*p* = 0.02).

**Figure 1:**
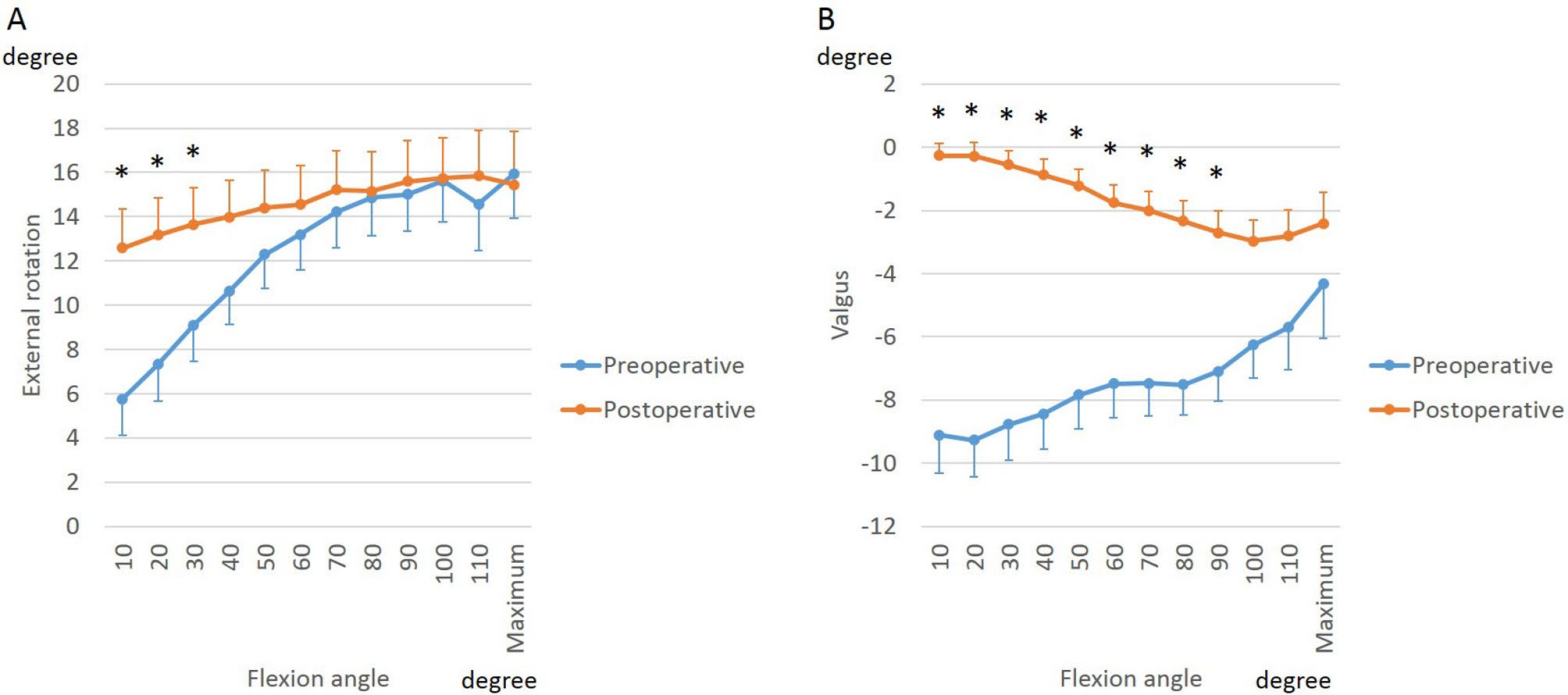
Effect of BCR-TKA on knee kinematics: external rotation (**A**) and valgus (**B**) of femoral component relative to tibial component.

**Figure 2:**
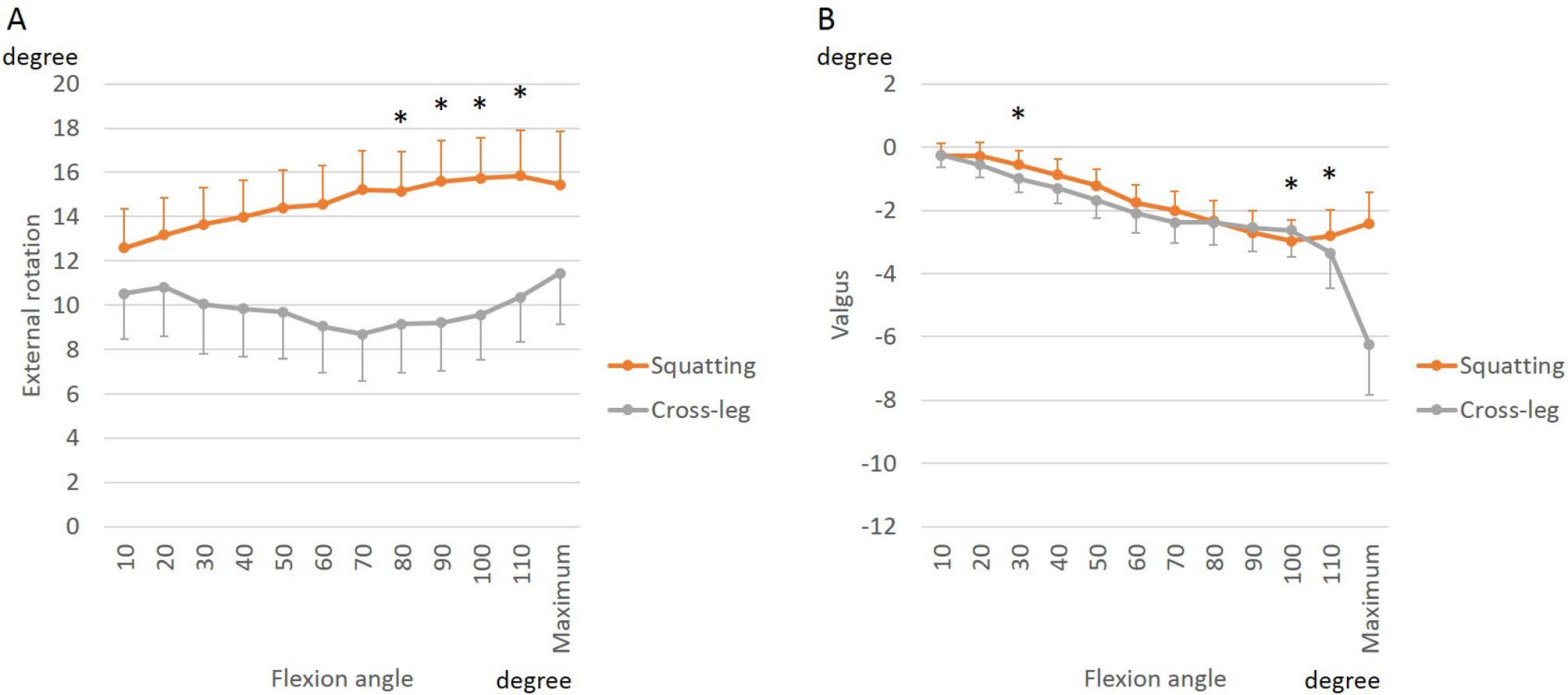
Differences in knee kinematics between squatting and cross-legged sitting: external rotation (**A**) and valgus (**B**) of femoral component relative to tibial component.

During squatting, the femur rotated externally relative to the tibia before and after BCR-TKA (Fig. 1A). The net range of external rotation of the femur reduced from 9.7 ± 4.8° preoperatively to (2.3 ± 4.8°) after BCR-TKA. In early flexion, the BCR femoral component was more externally rotated relative to the preoperative femoral rotation. During postoperative cross-legged sitting, relative to the rotation in extension, the femur rotated internally up to 70° of flexion, followed by external rotation beyond 70° of flexion. From 80° to 110° of flexion, the femoral external rotation during squatting was greater than that during cross-legged sitting (Fig. 2A).

BCR implantation also significantly changed varus-valgus angulation during the squatting activity (Fig. 1B). In extension, the varus angulation reduced from an average of 9.1 ± 4.5° to 0.3 ± 1.5°. BCR implantation also affected the change in varus angulation with flexion. With further flexion, preoperatively, the knees angulated toward less varus; while postoperatively, the knees angulated toward greater varus. The dynamic range of varus-valgus angulation was also significantly different: decreasing from a total range of 3.9 ± 4.4° preoperatively to 2.2 ± 2.7° postoperatively. There were minimal differences in varus-valgus angulation with cross-legged sitting except at maximum flexion that resulted in greater varus angulation than squatting (Fig. 2B).

### AP translation

**Figure 3:**
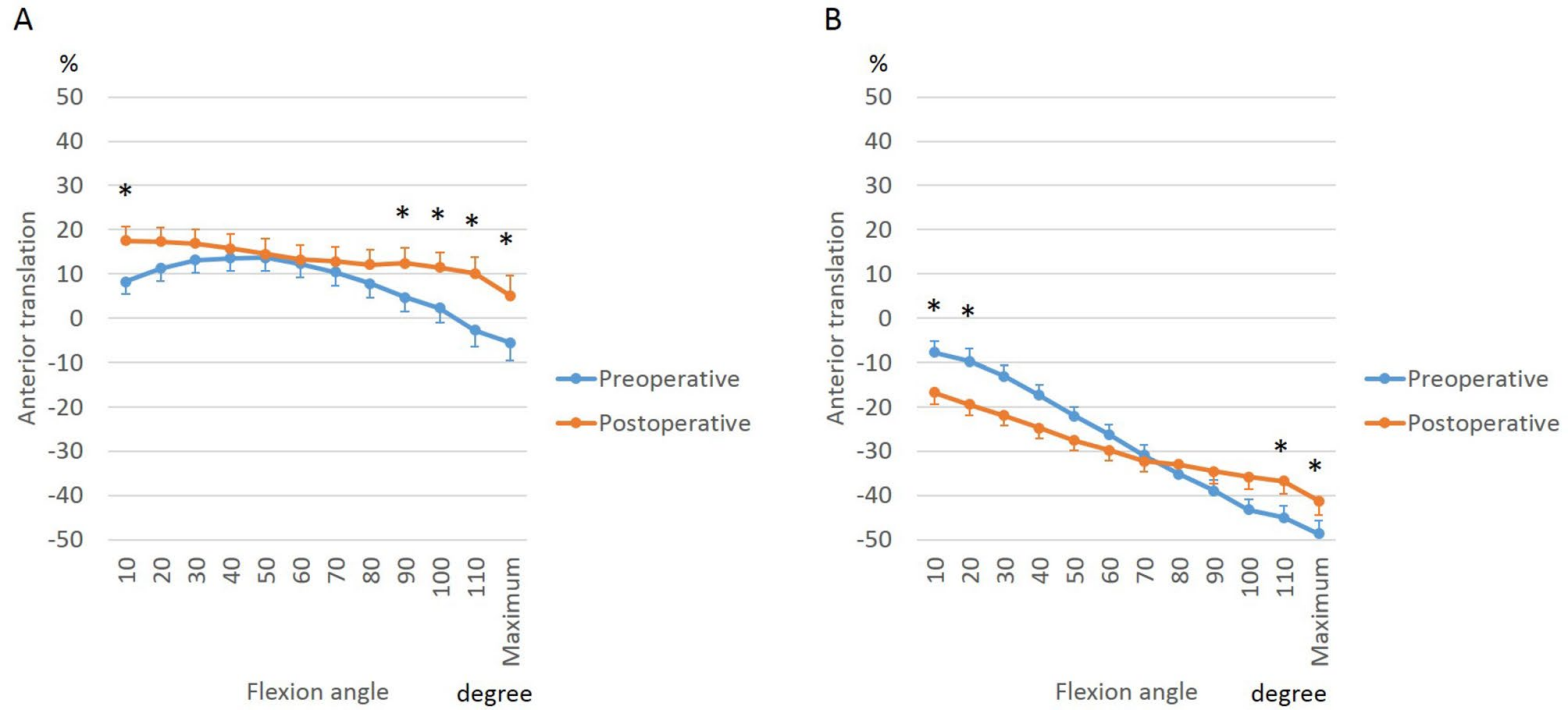
Effect of BCR-TKA on femoral AP translation of medial (**A**) and lateral (**B**) femoral condyle during squatting.

**Figure 4:**
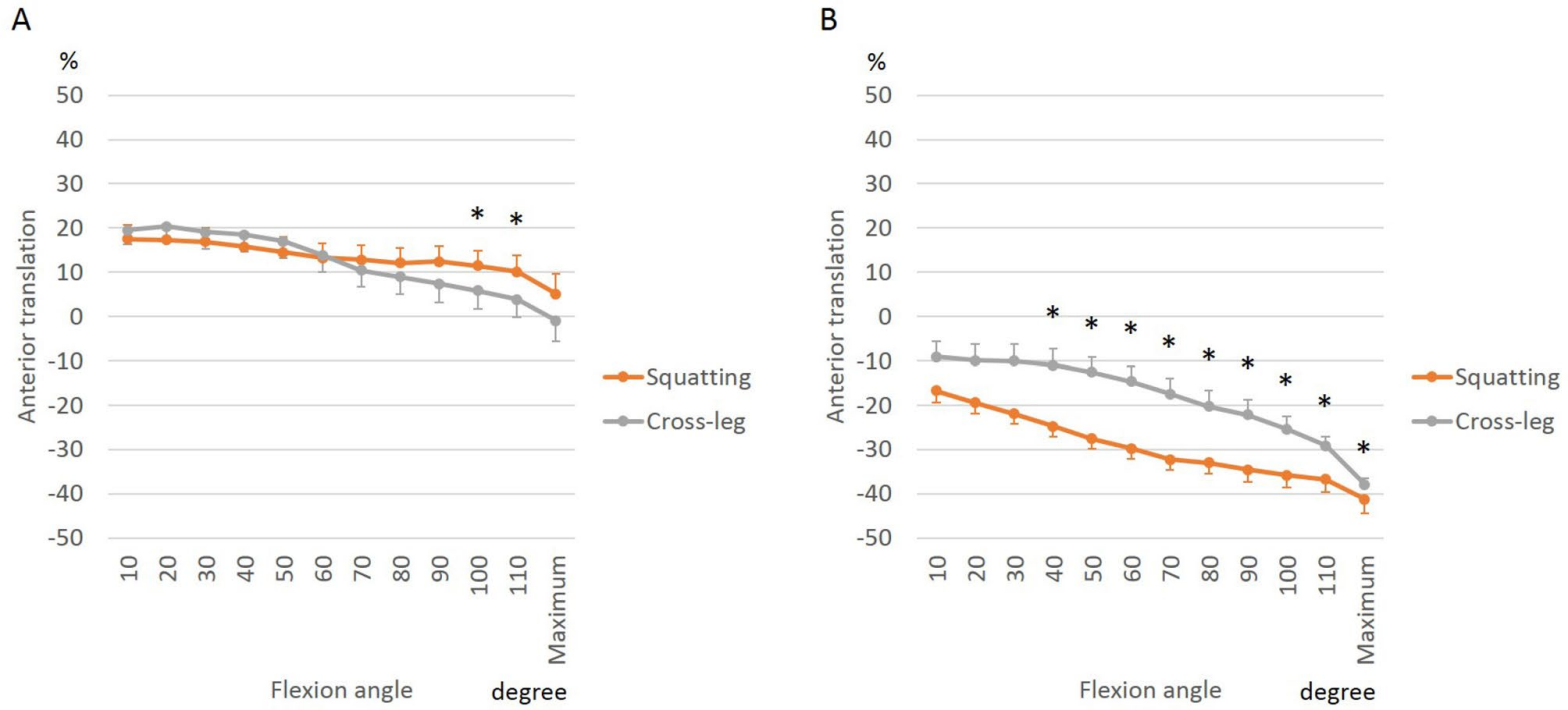
Differences between femoral AP translation of medial (**A**) and lateral (**B**) femoral condyle between squatting and cross-legged sitting.

To analyze femoral rollback and to identify the pivot pattern, we tracked the AP positions of the medial and lateral femoral condyles relative to the proximal tibial plane (Figs. 3 and 4). The relative translation of the femoral condyles is reflected in the shape of the curves in Figs. 3 and 4. While squatting preoperatively, the medial femoral condyle translated further anteriorly from 10° to 50° of flexion (3.9 ± 9.8%), and changed towards relative posterior translation beyond 50° of flexion (17.0 ± 8.2%). On the other hand, postoperatively, the medial femoral condyle translated relatively posteriorly throughout flexion for a total of 12.0 ± 10.9%. The net translation was computed as the difference between AP position in extension and that in flexion. Sitting cross-legged resulted in greater net posterior translation of the medial femoral condyle (total of 17.9 ± 11.4% posterior translation) than squatting (Fig. 4A).

The lateral femoral condyle translated posteriorly with flexion resulting in a medial pivot pattern, although the net posterior translation reduced postoperatively (range of 41.1 ± 10.5%, preoperatively; 21.9 ± 7.1% postoperatively, Fig. 3B). While squatting, the femur was more posteriorly located than during cross-legged sitting from 40° to maximum flexion (Fig. 4B).

### Cruciate ligament forces

**Figure 5:**
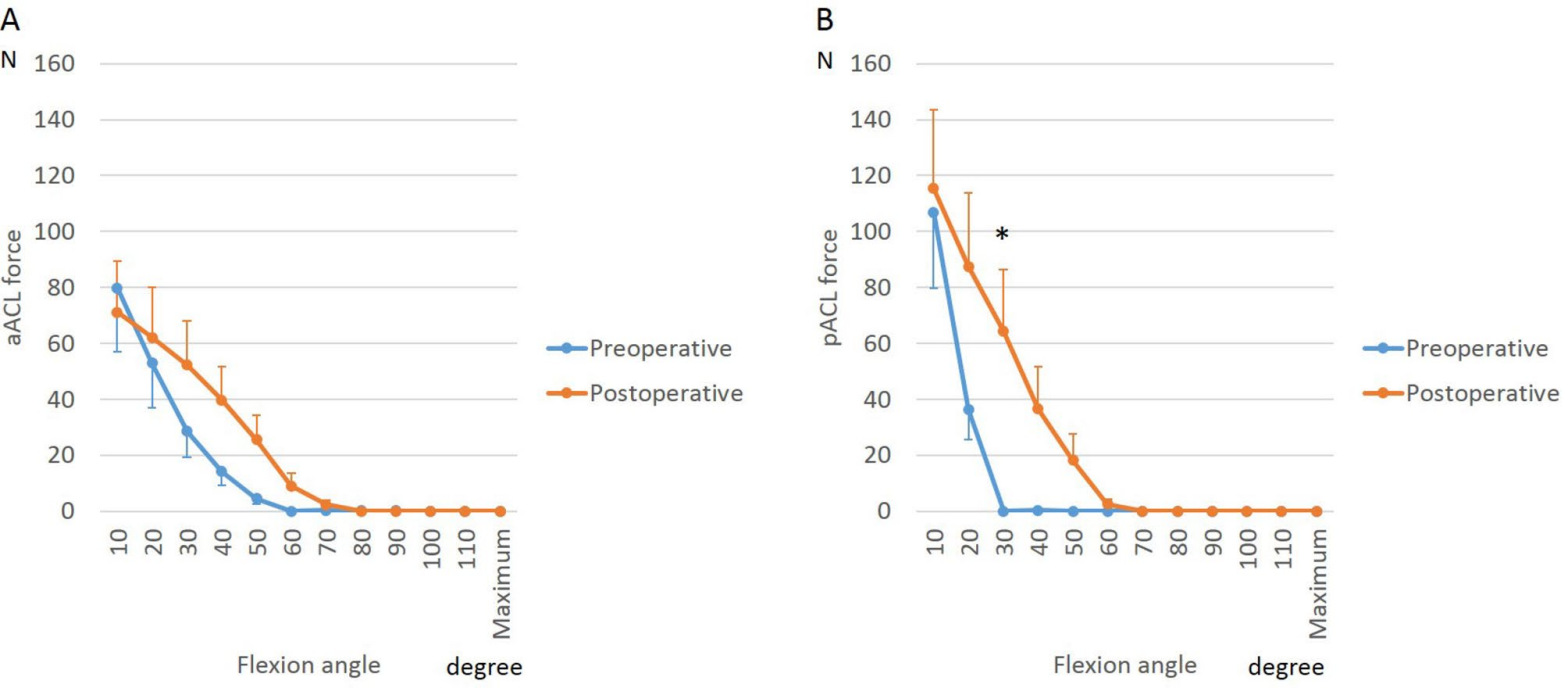
Anterior cruciate ligament (ACL) force during squatting before and after bicruciate-retaining total knee arthroplasty: (**A**) Anteromedial bundle of the ACL (aACL); (**B**) Posterolateral bundle of the ACL (pACL). *A significant difference between preoperative knees and postoperative knees (*p* ≤ 0.05).

**Figure 6:**
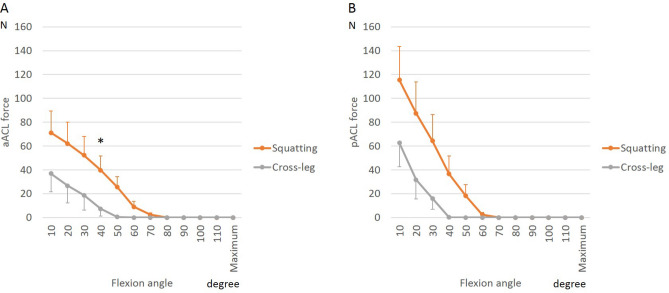
Anterior cruciate ligament (ACL) force during postoperative activities in bicruciate-retaining total knee arthroplasty: (**A**) Anteromedial bundle of the ACL (aACL); (**B**) Posterolateral bundle of the ACL (pACL). *A significant difference between knees during squatting and knees during cross-legged sitting (*p* ≤ 0.05).

To analyze the effect of BCR design on cruciate ligaments, we calculated tensile forces generated during squatting and cross-legged sitting. Tension in both bundles of the ACL was highest in extension preoperatively and remained high postoperatively. Postoperatively, however, at 30° of flexion, greater forces were generated in the pACL (*p* = 0.04, Fig. 5). Squatting generated greater tension in the aACL bundle than did cross-legged sitting at 40° of flexion (Fig. 6, *p* = 0.04).

**Figure 7:**
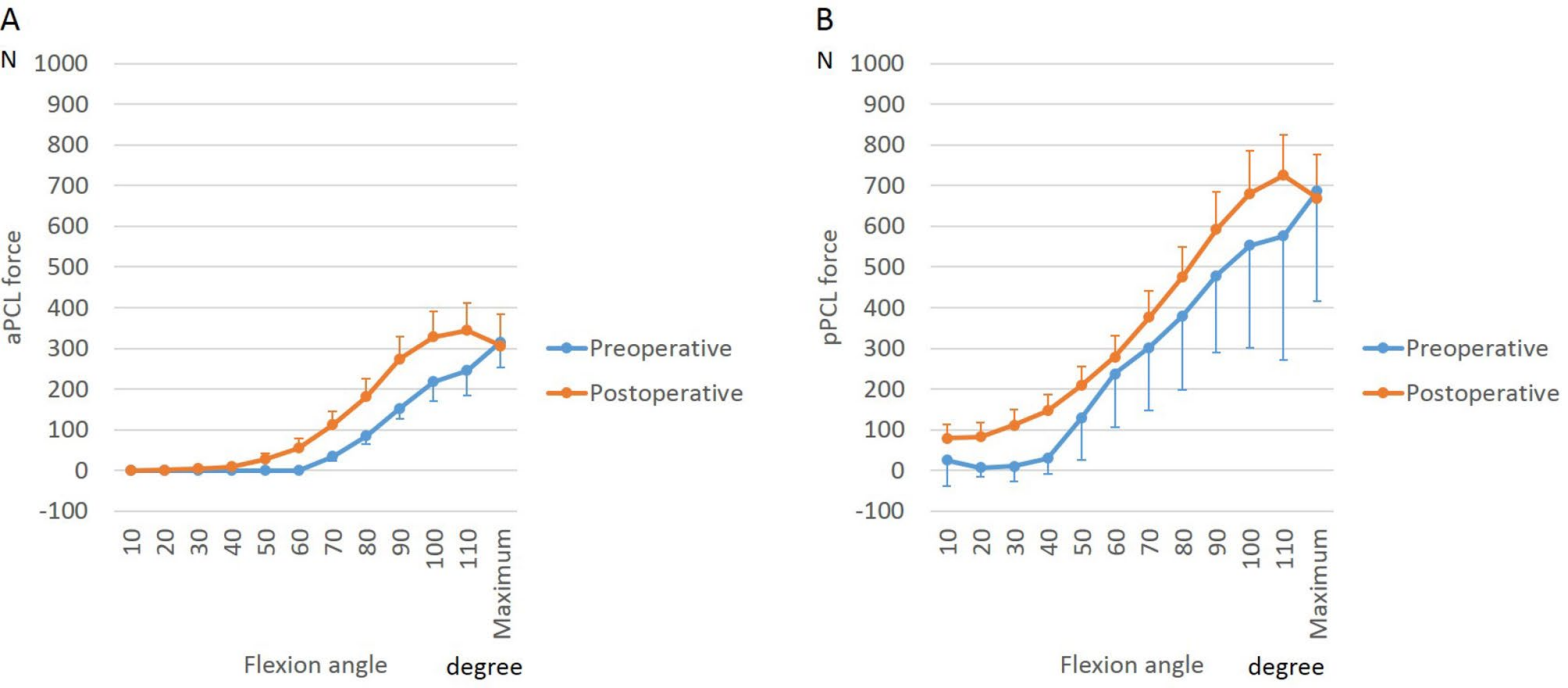
Posterior cruciate ligament (PCL) force during squatting before and after bicruciate-retaining total knee arthroplasty: (**A**) Anterolateral bundle of the PCL (aPCL); (**B**) Posteromedial bundle of the PCL (pPCL).

**Figure 8:**
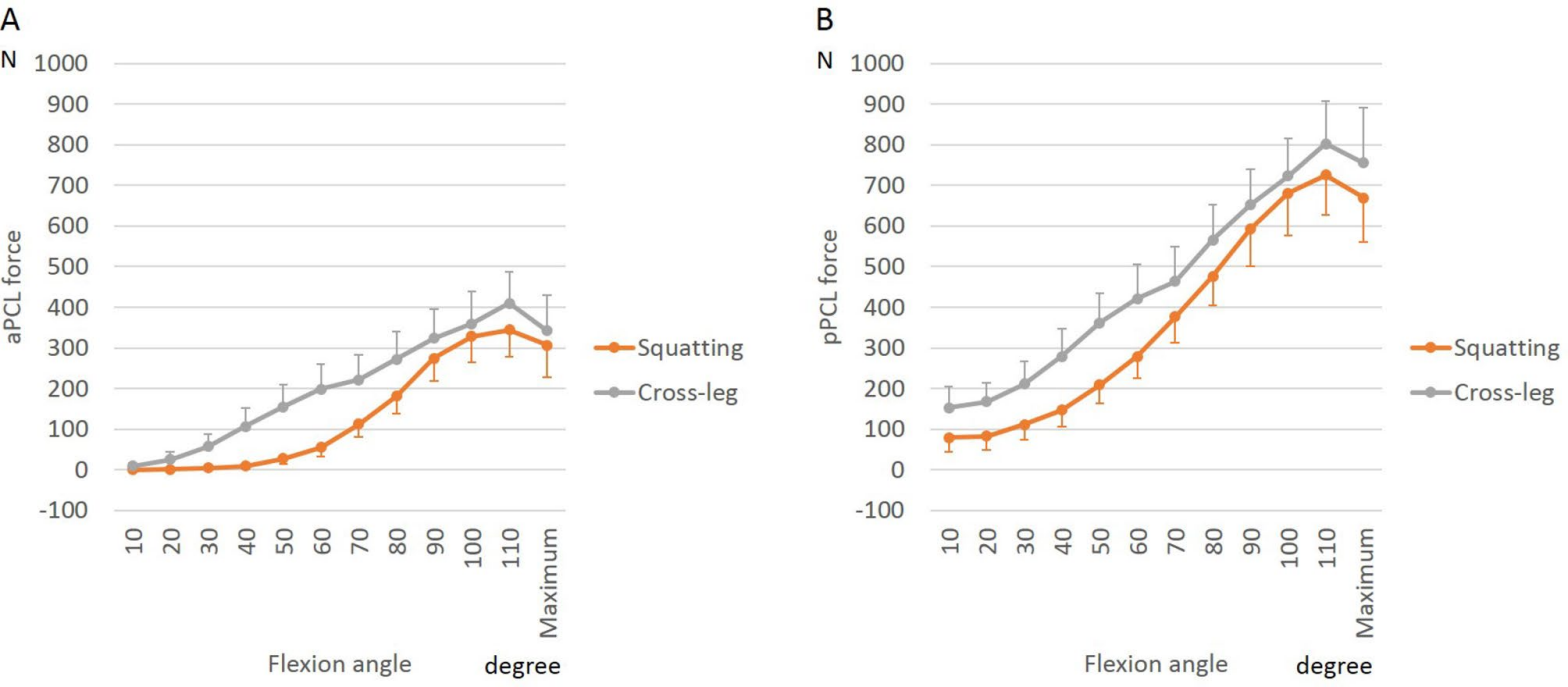
Posterior cruciate ligament (PCL) force during postoperative activities in bicruciate-retaining total knee arthroplasty: (**A**) Anterolateral bundle of the PCL (aPCL); (**B**) Posteromedial bundle of the PCL (pPCL).

In contrast to the ACL, tension in both PCL bundles increased with flexion and did not change significantly postoperatively, or with activity (Figs. 7 and 8).

### Correlation between the kinematics and cruciate ligament force

We used linear regression to determine whether knee kinematic patterns were associated with ligament tensile forces (Table 2). Preoperatively the ACL forces in both bundles correlated highly with the AP translation of the medial and lateral femoral condyles during squatting. Whereas, postoperatively the PCL forces correlated highly AP translation of lateral femoral condyle during postoperative squatting.

**Table 2:** Correlation between differences in knee kinematics and cruciate ligament force for each activity.

	Pre-squatting		Post-squatting		Post-cross-legged	
	Correlation coefficient	*p*-value	Correlation coefficient	*p*-value	Correlation coefficient	*p*-value
**aACL**						
Rotation angle	− 0.09	0.74	0.29	0.29	0.43	0.11
Varus-valgus angle	0.24	0.39	− 0.31	0.26	− 0.22	0.43
Medial AP translation	− 0.59	0.02	0.07	0.81	0.27	0.32
Lateral AP translation	− 0.71	≤ 0.01	− 0.23	0.40	− 0.22	0.43
**pACL**						
Rotation angle	0.14	0.62	0.42	0.12	0.23	0.41
Varus-valgus angle	− 0.14	0.62	− 0.39	0.15	0.21	0.46
Medial AP translation	− 0.69	≤ 0.01	− 0.09	0.75	0.08	0.77
Lateral AP translation	− 0.79	≤ 0.01	− 0.22	0.43	− 0.04	0.87
**aPCL**						
Rotation angle	− 0.14	0.61	− 0.42	0.12	− 0.12	0.66
Varus-valgus angle	0.50	0.06	− 0.01	0.96	0.09	0.75
Medial AP translation	0.20	0.47	0.04	0.88	0.24	0.39
Lateral AP translation	0.37	0.17	0.72	≤ 0.01	0.38	0.17
**pPCL**						
Rotation angle	0.15	0.61	− 0.13	0.66	− 0.15	0.59
Varus-valgus angle	0.31	0.26	− 0.33	0.23	0.13	0.65
Medial AP translation	0.35	0.20	0.15	0.59	0.18	0.51
Lateral AP translation	0.31	0.26	0.51	0.05	0.39	0.15

aACL: from 10° to 60° flexion; pACL: from 10° to 30° flexion; aPCL: from 60° to maximum flexion; pPCL: from 30° to maximum flexion.

## Discussion

We analyzed the effects of bicruciate-retaining total knee arthroplasty (BCR-TKA) on knee kinematics and cruciate ligament forces in order to identify changes induced postoperatively and to correlate knee kinematics with forces in the cruciate ligaments. The most important findings of this study were that (1) BCR-TKA reduced femoral external rotation and AP translation with flexion; (2) Preoperatively, ACL forces correlated highly with AP translation of the femur during squatting; and (3) Postoperatively, only PCL forces correlated highly with AP translation of the lateral femoral condyle during squatting.

OA of the knee significantly affects knee kinematics^1,2,18^. These kinematic changes were not fully restored after traditional ACL-sacrificing surgery^3^. While BCR designs have been developed to retain the ACL, BCR-TKA also does not restore normal knee kinematics^13,19,20^. Our previous study demonstrated that knees after BCR-TKA with an anatomical articular surface showed lower femoral external rotation and lateral posterior translation with flexion than normal knees during squatting^19^. Normal knees during squatting displayed large femoral external rotation at early-flexion called screw-home movement^21,22^, and medial-pivot motion followed by femoral rollback^22^. In a cadaveric study, Hamada et al. reported that the screw-home movement of the intact knee was retained after initial implantation of the BCR-TKA femoral component but was lost after the BCR-TKA tibial component was implanted^23^.

Knee kinematic patterns are highly relevant to clinical outcomes. Van Onsem et al. reported that patients with a poor patient-reported outcome measure (PROM) after TKA experience more anterior translation on the medial side followed by a medial mid-flexion instability and less posterior translation on the lateral side in high-flexion than patients with good PROM scores^24^. Twiggs et al. reported a varus angular change from 10° to 90° flexion of between 0° and 4° had a significant improvement in postoperative KOOS pain score in cruciate-retaining TKA (CR-TKA)^25^. In our study, BCR-TKA eliminated the anterior translation of the medial condyle, preserved the medial pivot pattern, and corrected the preoperative varus angulation. Improvement in the positive features of knee kinematics during high flexion activities may improve PROM scores after BCR-TKA.

ACL forces were high only in the early range of flexion. Preoperatively, posterior translation of the femur positively correlated with higher ACL force during squatting. Postoperatively, the high ACL force in early flexion and consistent posterior femoral translation during flexion indicated that the ACL was actively providing stability against paradoxical anterior translation. Okada et al. found the ACL in situ force against 100 N of anterior force in BCR-TKA knees to be statistically comparable with that in intact knees at all flexion angles^13^. Furthermore, Sabouret et al. reported that ACL-retaining TKA remained functional and provided adequate stability at the 22-year follow-up^26^. These results support the positive influence of ACL retention in BCR-TKA on clinical outcomes.

Forces in the PCL increased with flexion and posterior translation of the lateral femoral condyle correlated with reduced PCL force during postoperative squatting. Tsai et al. reported that the PCL in BCR-TKA with symmetrical articular surfaces was significantly overstretched in deep flexion positions^27^. These results were similar to a prior study of CR-TKA^28^, which also reported overstretching of the PCL in CR TKA during deep flexion. This overstretching was attributed to reduced femoral rollback secondary to kinematic conflict. The BCR-TKA design used in our study had an anatomical articular surface, which presumably reduced kinematic conflict and reduced the potential for overstretching of the PCL.

The type of high-flexion activity also had an effect on knee kinematics. Squatting induced greater femoral external rotation and cross-legged sitting generated more varus angulation at maximum flexion, as we have previously reported^17^. In addition, this kinematic difference, depending on the type of high-flexion activity, was similar to that of normal knees^22^. ACL forces during cross-legged sitting also tended to be lower than those during squatting. Henning et al. reported significant differences in ACL strain between closed-kinetic-chain and open-kinetic-chain activities^29^. Therefore, different types of high-flexion activities are likely to have distinct effects on ACL forces.

This study has some limitations. First, the study did not directly compare BCR-TKA with ACL-sacrificing designs. However, the kinematics of ACL-sacrificing designs have been extensively documented. Second, the study cohort had a relatively short mean follow-up duration of 8.1 months. Kinematics and ligament forces at longer-term follow-up may differ from those of this study.

This is the first study to report in vivo ligament forces before and after BCR-TKA. BCR-TKA resulted in significant changes in knee kinematics during deep knee bend activities. These kinematic changes correlated significantly with changes in cruciate ligament forces. These results are valuable to develop appropriate ligament balancing strategies and to enhance and develop BCR knee designs.

## Methods

A total of 15 knees in 13 patients (3 males and 10 females), who underwent BCR-TKA (Journey II XR, Smith & Nephew, Memphis, TN, USA), were enrolled in this study. The patients provided informed consent to participate in the study after institutional review board approval (provided by The University of Tokyo Institutional Ethics Review Board). The following methods were carried out in accordance with relevant guidelines and regulations.

Knee kinematics were recorded fluoroscopically while each patient performed a closed-kinetic chain deep knee bend (squatting) and an open-kinetic chain high flexion activity (sitting cross-legged)^17^. Preoperative kinematics (within one month of surgery) were recorded during squatting. Postoperative kinematics (at least six months after BCR-TKA) were recorded during squatting and sitting cross-legged. Kinematics during cross-legged sitting were not recorded preoperatively, as most patients found it painful to perform this activity. The activity was performed at a natural pace under fluoroscopic surveillance in the sagittal plane. The mean duration of postoperative follow-up was 8.1 ± 8.2 months. At postoperative fluoroscopic analysis, the mean age was 72.3 ± 5.9 years. The mean body height was 157.4 ± 6.9 cm and the mean body weight was 60.2 ± 7.9 kg. All patients underwent BCR-TKA to treat bicompartmental or tricompartmental OA with an intact ACL. Presence of an intact ACL was confirmed using preoperative magnetic resonance imaging (MRI). The Knee Injury and Osteoarthritis Outcome Scores (KOOS)^30,31^ and hip-knee-ankle angle (HKA) were recorded pre- and postoperatively (Table 1). The sequential motion was recorded as a series of digital X-ray images (1024 × 1024 × 12 bits/pixel, 7.5-Hz serial spot images as a DICOM file) using a 17-inch (43-cm) flat panel detector system (ZEXIRA DREX-ZX80, Toshiba, Tokyo, Japan). Furthermore, all images were processed by dynamic range compression, thereby enabling edge-enhanced images. To estimate the spatial position and orientation of the knee automatically, a two-dimensional/three-dimensional (2D/3D) registration technique^32^ was employed based on a contour-based registration algorithm using single-view fluoroscopic images and 3D computer-aided design (CAD) models. The estimated accuracy of relative motion between metal components was ≤ 0.5° in rotation and ≤ 0.4 mm in translation^32^.

**Figure 9:**
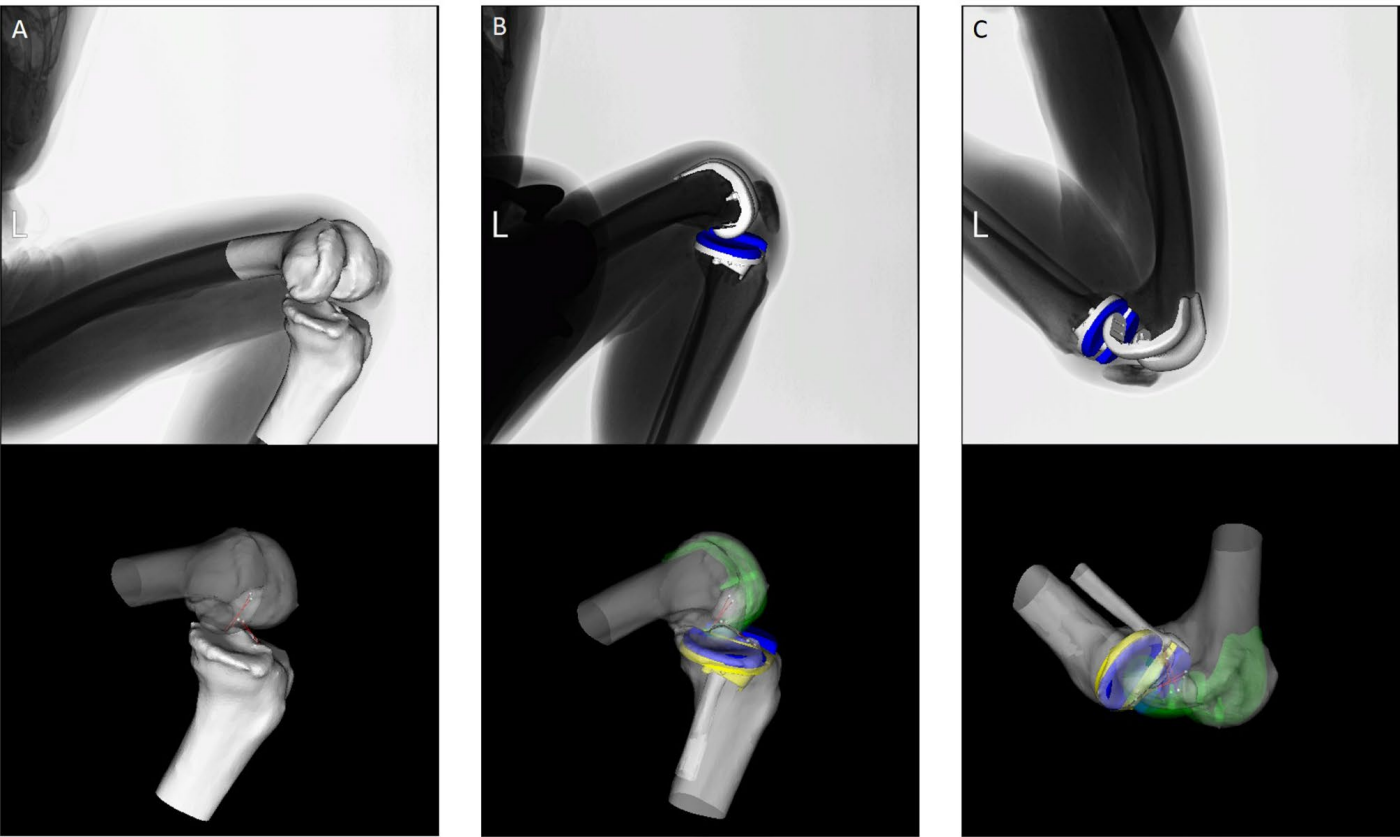
A two-dimensional/three-dimensional (2D/3D) registration and the cruciate ligament force analysis in bicruciate-retaining total knee arthroplasty (images generated by visualization software developed at Osaka University, Osaka, Japan). Red lines at the bottom row indicate the cruciate ligament models: (**A**) Preoperative squatting; (**B**) Postoperative squatting; (**C**) Postoperative cross-legged sitting.

We used the same local coordinate system for preoperative and postoperative analysis by constructing 3D bone models from preoperative and postoperative computed tomography (CT) scans. In BCR-TKA knees, extraction of the bony contour creation was difficult because of metal artefact in the CT scans. Thus, 2D/3D registration of femoral and tibial implants was performed initially; subsequently 2D/3D registration of femoral and tibial bone models was performed as previously reported^19^. The relative position between the implant and the bone was matched using surface registration between preoperative 3D bone models and 3D implant and bone models created from postoperative CTs (Fig. 9)^19^. Estimated accuracy for the relative motion between the femoral and tibial 3D bone models was ≤ 1° in rotation and ≤ 1 mm in translation^22^. The local coordinate system for the femur and tibia was constructed as previously described^22^. Knee rotations were described using the joint rotational convention of Grood and Suntay^33^.

The femoral and tibial attachment areas of the anteromedial and posterolateral bundles of the ACL (aACL and pACL), and the anterolateral and posteromedial bundles of the posterior cruciate ligament (aPCL and pPCL) were identified using the osseous landmark from preoperative CT and MRI data)^34–36^. The accuracy of the attachment area is 0.7 ± 0.1 mm^36^. Tensile forces in each cruciate ligament bundle were calculated using commercially available software (VivoSim v1, Advanced Mechanical Technology Incorporated, Watertown, MA, USA; https://amti.biz/index.aspx). The path of each ligament was approximated as a straight line (Fig. 9); the effects of ligament-bone contact were neglected. VivoSim computes tensile forces in each ligament bundle based on the strain calculated by the relative movement of the bony attachments points during knee flexion. Tensile force is computed for each bundle using properties described by a nonlinear force–strain curve^37–39^. The parameters used (initial stiffness values and reference lengths of the ligament bundles) were based on the data reported by Shelburne et al^39,40^. The properties of the model ligaments were adapted to match measurements of knee-joint laxity in the intact and ACL-deficient knee obtained from previous cadaver studies^38,41^.

Femoral rotation and varus-valgus angle relative to the tibia, anteroposterior (AP) translation of the medial sulcus (medial side), and lateral epicondyle (lateral side) of the femur on the plane perpendicular to the tibial mechanical axis in each flexion angle were evaluated^22^. AP translation was calculated as a percentage relative to the proximal AP dimension of the tibia^19,22^. External rotation was denoted as positive; internal rotation as negative. Valgus angulation was defined as positive; varus as negative. Positive or negative values of AP translation were defined as anterior or posterior to the axis of the tibia, respectively. Cruciate ligament forces for each flexion angle were calculated. To investigate whether ligament forces were related to knee kinematics, for each activity, we computed the change in femoral rotation, varus-valgus angulation, and AP position of the femoral condyles and change in ligament forces for each ligament bundle for selected flexion ranges as follows: aACL: change from 10° to 60° flexion; pACL: change from 10° to 30° flexion; aPCL: change from 60° to maximum flexion; pPCL: change from 30° to maximum flexion. These ranges of flexion were selected because forces are more likely to be generated in the ACL and PCL within these ranges. We then evaluated the correlation between change in each kinematic parameter and change in force for each cruciate ligament bundle. All the values were expressed as mean ± standard deviation.

### Statistical analyses

Results were analyzed using SPSS version 24 (IBM Corp., Armonk, NY, USA). Repeated measure analysis of variance (ANOVA) and post hoc pairwise comparison (Bonferroni test) were used to analyse all evaluated items except the knee flexion angle, KOOS, HKA, and correlation. A paired t-test (parametric data) and Wilcoxon signed-rank test (non-parametric data) were used to compare knee flexion angle and each subscale in KOOS and HKA. Pearson’s Correlation Coefficient was used to analyze the correlation between differences in knee kinematics and corresponding differences in cruciate ligament force. A *p*-value ≤ 0.05 was considered statistically significant. Power analysis using G*Power 3.1^42^ indicated that nine knees would be required to generate a power at 0.8 at an alpha of 0.05.
